# Impact of *Plasmodium falciparum* infection on haematological parameters in children living in Western Kenya

**DOI:** 10.1186/1475-2875-9-S3-S4

**Published:** 2010-12-13

**Authors:** Robert N Maina, Douglas Walsh, Charla Gaddy, Gordon Hongo, John Waitumbi, Lucas Otieno, David Jones, Bernhards R Ogutu

**Affiliations:** 1 US Army Medical Research Unit-Kenya (Walter Reed Project), Nairobi, Kenya; 2 Centre for Clinical Research, Kenya Medical Research Institute, Nairobi, Kenya; 3 USAMRU-K (Walter Reed Project), Centre for Clinical Research, Kenya Medical Research Institute, P.O.Box 54- 40100, Kisumu, Kenya

## Abstract

**Background:**

Malaria is the commonest cause of childhood morbidity in Western Kenya with varied heamatological consequences. The t study sought to elucidate the haemotological changes in children infected with malaria and their impact on improved diagnosis and therapy of childhood malaria.

**Methods:**

Haematological parameters in 961 children, including 523 malaria-infected and 438 non-malaria infected, living in Kisumu West District, an area of malaria holoendemic transmission in Western Kenya were evaluated.

**Results:**

The following parameters were significantly lower in malaria-infected children; platelets, lymphocytes, eosinophils, red blood cell count and haemoglobin (Hb), while absolute monocyte and neutrophil counts, and mean platelet volume (MPV) were higher in comparison to non-malaria infected children. Children with platelet counts of <150,000/uL were 13.8 times (odds ratio) more likely to have malaria. Thrombocytopaenia was present in 49% of malaria-infected children and was associated with high parasitaemia levels, lower age, low Hb levels, increased MPV and platelet aggregate flag. Platelet aggregates were more frequent in malaria-infected children (25% vs. 4%, p<0.0001) and associated with thrombocytopaenia rather than malaria status.

**Conclusion:**

Children infected with *Plasmodium falciparum* malaria exhibited important changes in some haematological parameters with low platelet count and haemoglobin concentration being the two most important predictors of malaria infection in children in our study area. When used in combination with other clinical and microscopy, these parameters could improve malaria diagnosis in sub-patent cases.

## Background

Blood is the most easily accessible diagnostic tissue. Changes in haematological parameters are likely to be influenced by any disease condition which affects the haemopoetic physiology at any level. This is likely to happen with an endemic disease such as malaria that affects the host homeostasis at various fronts resulting in a myriad of clinical presentation. Malaria is a major cause of morbidity in the tropics. Two hundred and forty seven million cases were reported worldwide in 2006 [1]. Haematological changes are some of the most common complications in malaria and they play a major role in malaria pathology. These changes involve the major cell lines such as red blood cells, leucocytes and thrombocytes. In Western Kenya, severe anaemia is the predominant severe malaria syndrome peaking in the first two years of life and is attributed to *Plasmodium falciparum*[2]. In malaria-infected patients, especially non-immunes children, prompt and accurate diagnosis is key to effective disease management for a favourable outcome. Clinical diagnosis is widely used for diagnosis of malaria especially in resource-poor countries. Although fever and other signs and symptoms are known to be fairly sensitive measures of malaria they lack specificity and positive predictive values especially in areas where malaria is less prevalent [3-6]. This is likely to be the scenario with changing epidemiology of malaria in Africa. Moreover, in tropical countries where malaria is most prevalent, it may be difficult to distinguish the disease from other infections e.g. viral or bacterial based on the symptoms and signs [6,7]. Presumptive anti-malarial treatment is widely practiced and studies show that it is wrought with significant misuse of anti-malarial drugs [4,8]. Microscopic diagnosis is the ‘’imperfect gold standard” for malaria parasite detection and speciation. This technique requires technical expertise and is time-consuming in repeated smear examinations [6]. However, it is a valuable technique when performed correctly in the right hands but can be unreliable and perceived as wasteful when poorly executed [7-9]. Haematological changes in malaria, such as anaemia, thrombocytopaenia and leucocytosis or leucopaenia are well recognized. The extent of these alterations varies with level of malaria endemicity, background haemoglobinopathy, nutritional status, demographic factors, and malaria immunity [3,10,11]. Furthermore, diagnostic value of these haematological alterations has not been established in children living in malaria endemic areas. The present study examines the occurrences and severity of haematological changes and their diagnostic value in children with *P. falciparum* malaria in Kisumu, western Kenya. Haematological parameters (red blood cells, white blood cells, platelets, red cell distribution width, mean platelet volume and haemoglobin) of children less than 5 years infected with *P. falciparum* are compared with uninfected children from the same community. In this study, haematological patterns and their possible predictive values of malaria infection are identified.

## Methods

This study was approved by Kenya Medical Research Institute (KEMRI) Scientific Steering Committee and Ethical Review Committee, Walter Reed Army Institute of Research (WRAIR) Scientific Review Committee, US Army Medical Research and Materiel Command (USAMRMC), Human Subjects Research Review Board (HSRRB), and the PATH Human Subject Protection Committee (HSPC). The parents/guardians of the study participants gave a written informed consent before being enrolled into the study. The consent included long-term secure storage of collected samples and information generated during the study and their use for research. The study was conducted at the Kombewa, Kenya Medical Research Institute – US Army Medical research Unit-Kenya (KEMRI/USAMRU-K) Clinical Trails Unit in Kisumu West District of western Kenya an area of intense malaria transmission. The data used in this study were collected during the screening and follow-up phases of a malaria vaccine trial in March – December 2005 [12]. The study was reviewed and approved by the Kenya Medical Research Institute’s ethical review committee.

Laboratory records of children enrolled in the vaccine trial were reviewed. Nine hundred and sixty one children (age <5yrs) were included in this analysis. This included 523 children diagnosed with *P. falciparum* malaria and 438 non-malaria infected children from the same community.

The investigations were performed on venous blood sample drawn into EDTA tubes for preparation of the thick and thin smears for malaria parasites and automated determination of Complete Blood Counts (CBCs). Blood counts were performed using ACT5 Diff Haematology Analyzer (Beckman Coulter Inc, Miami, Florida, USA) as per local SOPs within 1 hour.

Daily Quality Assurance checks were performed and recorded; commercial controls were used in accordance with manufacturer’s recommendations. The Analyzer provided data on WBCs, RBCs, haemoglobin level, platelet counts, Mean platelet volume, red cell distribution width (RDW) and five part differentials. The Analyzer also detected and flagged as platelet aggregation and cold agglutinins based on the particle size using a 256-channel pulse–height analyzer of platelet histogram region.

Two blood slides were prepared and stained with Giemsa. One slide from each study participant was examined independently by two experienced microscopists. They determined presence or absence of malaria parasites, the species and the number of asexual parasites. Parasite enumeration was performed on thick film/200 WBCs or thin film/2,000 RBCs, depending on the parasite density. Results of the two primary readers were averaged if concordant and used for the calculation of parasite density. Non-concordance in species or parasite counts between the primary readers was referred to a third microscopist (tie breaker) whose determination of species and parasite count was considered final. Parasite densities were calculated as parasite/µL of blood (parasite/WBCs counted x total WBCs in a µL of blood or parasite/RBCs counted x total RBCs in a µL of blood). Percent parasitaemia was calculated as follows; parasite count per µL of blood/RBCs per µL x 100. These blood samples were also used for determining the presence of haemoglobinopathies (α-thalassaemia trait, G6PD status and Hb type).

## Data analysis

Data analysis was performed using GraphPad prism software (GraphPad software, Inc, San Diego Calif.). Normally distributed continuous data were compared using student’s t-test with 2 tailed *P*-values, whereas data not conforming to normal distribution were compared by Mann-Whitney U test. Categorical variables were compared using Fisher’s exact test. Association between two continuous variables was assessed by Spearman’s rank correlation. Diagnostic accuracy of haematological parameters was measured by computing sensitivity, specificity, predictive values and odds ratios. Precision of these parameters was evaluated using 95% confidence intervals.

## Results

Nine hundred and sixty one children were included in this study. The mean age was 32 months (5-48) and 56% were males. Fifty four percent (n=523) of the subjects had *P. falciparum* malaria confirmed by microscopy while the remaining were negative and were used as controls.

The mean values of selected haematological parameters for the non-malaria infected group (controls) were determined for this population stratified by sex. T-test showed no significant difference in Hb, platelets, total WBCs, RDW, and RBC counts between males and females. As a result, subsequent analysis of continuous variables was not stratified by sex.

Haematological parameters of the malaria parasitemic group were compared with those of the controls using t-test (Mann-Whitney U test). Median values for Haemoglobin, Platelet count, RBC count lymphocyte and eosinophils counts were significantly lower for the parasitemic group compared with the controls. Conversely, the Mean platelet volume (MPV), Monocyte and neutrophil counts were significantly higher in the parasitemic group. There was no significant difference in total WBC and RDW between the parasitemic and the non-parasitemic groups (Table 1).

**Table 1:** Haematological values in children with positive

***Variable***	***Malaria, n=523***	***No malaria, n=438***	***P- Value***
	**Mean** **(Median)**	**Mean** **(Median)**	
Age (months)	31.2 (24)	31.6 (24)	0.72
Hemoglobin (g/dl)	9.5 (9.2)	10.9 (10.8)	<0.0001
Platelets (x10^3^/μL)	195 (174)	334 (312)	<0.0001
RDW (%)	16.4 (15.9)	16.7 (16.4)	0.15
MPV (fl)	8.43(8.4)	7.65 (7.7)	<0.0001
WBC(x10^3^/μL)	11.2 (10)	10.4 (9.1)	0.27
Lymphocytes (x10^3^/μL)	4.7. (4.2)	5.3 (4.95)	0.0009
Monocytes (x10^3^/μL)	1.6 (1.3)	1.2 (1.02)	<0.0001
RBC (x10^6^/μL)	4.0 (4.1)	4.7 (4.6)	<0.0001
Neutrophils (x10^3^/μL)	3.8 (2.8)	3.3 (2.6)	0.0076
Eosinophils (x10^3^/μL)	0.2 (0.14)	0.4 (0.23)	0.0001

### Haemoglobin

Anaemia was defined as haemoglobin level <10g/dl for both males and females and further classified as severe if Hb <5g/dl. Severe malaria anaemia was consequently defined as Hb<5g/dl in the presence of hyperparasitaemia (> 200,000 parasites/μL) [13]. The median haemoglobin value of the malaria-infected group was significantly lower than the negative group (9.2g/dl vs. 10.8g/dl, *P*<0.0001). Three hundred and twelve (60%) of the malaria-infected children had anaemia with 18(3%) being severe compared to 118 (27%) in the non-malaria infected group all being mild anaemia. There was poor correlation between parasitaemia and haemoglobin (r=-0.08, *p =0.78)*.

Of the 112 anaemia cases in the non-malaria infected group, 42% had some type of haemoglobinopathy (HbAS=10%, G6PD deficiency=6% and mutant α-globulin gene=25%) compared to 25% (HbAS=6%, G6PD^-^=4% and α*=16%) of non anaemic children (Table 2). These cases of haemoglobinopathies are thought to have contributed to the anaemia observed in some of the non-malaria infected children. Haemoglobinopathy data for 310 subjects was not available. Hb level positively correlated with age (r=0.24, p=0.0005).

**Table 2:** Haemoglobin levels and distribution of haemoglobinopathies in the study groups

No Malaria					Malaria			
n=438					n=523			
Hb level	N	HbAS	HbAA	Unknown	n	HbAS	HbAA	Unknown
<5g/dl	0	0	0	0	7	0	1	6
5-10g/dl	29	12	10	4	70	24	12	34
>10g/dl	73	18	47	10	65	20	8	37

Hb level	N	G6PD-	G6PD	Unknown	N	G6PD^-^	G6PD	Unknown

<5g/dl	0	0	0	0	5	0	0	5
5-10g/dl	17	7	6	3	76	19	20	37
>10g/dl	48	12	31	6	85	8	35	42

Hb level	N	α*	α _2_α_1/_α _2_α_1_	Unknown	n	α*	a_2_a_1_/a_2_a_1_	Unknown

<5g/dl	0	0	0	0	6	0	1	5
5-10g/dl	72	30	31	9	131	68	8	55
>10g/dl	199	50	122	30	78	47	4	27

G6PD^-^ = deficient G6PD activity, G6PD= normal activity, αα/αα=normal α-globulin gene, α*=defective α-globulin gene

### Platelets

Thrombocytopaenia was defined as platelet count <150 x 10^3^/μL and further defined as severe if the platelet count <50 x 10^3^/μL. The median platelet count for the 523 children in the malaria-infected group was significantly lower than non- malaria infected group (174 x 10^3^/μL vs. 312 x 10^3^/μL; *p*<0.0001). Thrombocytopaenia was reported in 255 (49%) of the malaria-infected children with 25 (5%) being severe as compared to 16 (4%) in the non-malaria infected group which had no cases of severe thrombocytopaenia (Table 3).

**Figure 1:**
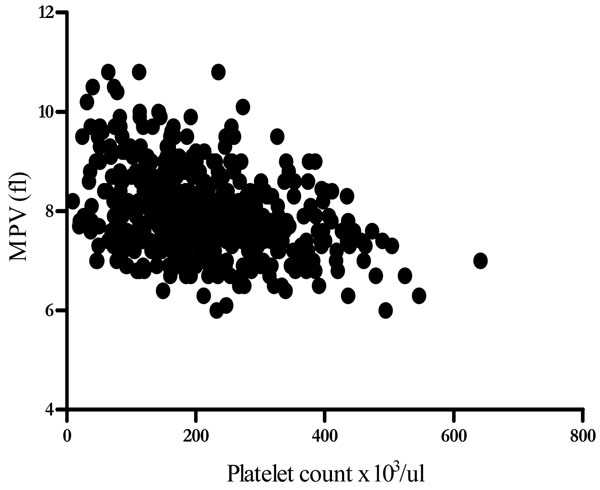
Relationship between Platelets counts and MPV

**Figure 2:**
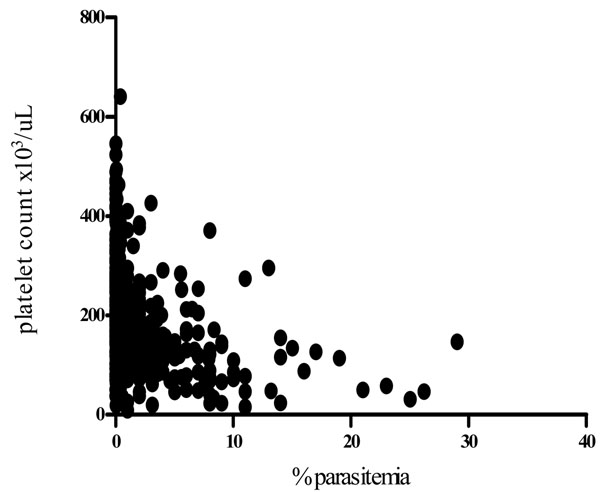
Relationship between Platelet count and Parasitaemia

Thrombocytopaenia was also weekly associated with, anaemia in the malaria infected group (r=0.2, *P=0.0003*) and age (r=0.14, *p=0.0073*). There was a strong inverse correlation between platelet counts and mean platelet volume (Figure 1). Mean MPV increased as platelet count fell in both malaria infected (r=-0.4, *p<0.0001*) and Non-infected (r=-0.34, *p<0.0001*) group. There was an inverse relationship between parasitaemia and platelet counts (r=-0.44, *p<0.0001)* (Figure 2). Over 50% of samples with a parasitaemia above 10% had a platelet count <50,000/μL. A decreasing trend of platelet counts across quartiles of parasite densities was also evident (Table 4).

**Table 3:** Distribution of platelet counts in the study groups

Platelet count	Malaria Positive N=523	Malaria Negative N=438
<50x10^3^/μL	25 (5%)	0
<50x10^3^/μL	230 (44%)	16 (4%)
151-450x10^3^/μL	258 (49%)	356 (81%)
>450x10^3^/μL	10 (2%)	66(15%)

**Table 4:** Distribution of platelet counts according to parasitaemia levels

Parasitemia level	N	<50x10^3^/μl	51-150x10^3^/μl	151-450 x10^3^/μl	>450x10^3^/μl	Mean platelet count x10^3^/μl
**≤1%**	312	3 (1%)	95(30%)	205 (66%)	9(3%)	235
**1.1-5.0%**	157	6(4%)	109(69%)	41 (26%)	1(1%)	187
**5.1-10%**	37	7(19%)	19(51%)	11(30%)	0%	145
**>10%**	17	9(53%)	7(41%)	1(6%)	0%	117
Total	523	25	230	258	10	

#### Platelet count and parasite density

Parasitaemia levels (%) ranged from 0.0004 to 29 with a mean of 1.9 (95% CI, 1.6-2.2). Most children (60%) had parasitaemia ≤1%, 157 (30%) had values between 1 - 5%, 37 (7%) had values between 5.1-10%, and 17 (3%) had a parasitaemia >10% (Table 4). Parasitaemia levels were not affected by gender (median males 1% vs. Females 1.3%, p=0.47). Percent parasitaemia was inversely associated with age (r=-0.2, p=0.0134) with younger children having higher parasite densities.

**Figure 3:**
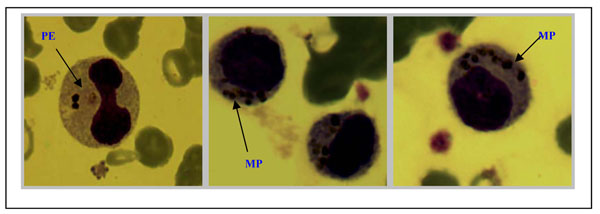
Monocytes containing malaria pigments (MP) and phagocytosis of parasitized erythrocytes (PE)

**Figure 4:**
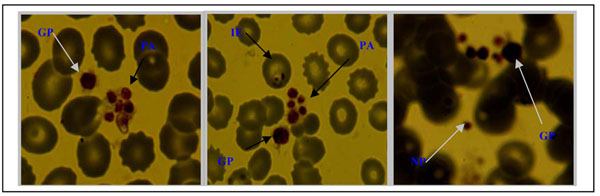
Small platelet aggregates (PA), giant platelets (GP), normal platelets (NP) and infected erythrocyte (IE) seen in anticoagulated blood.

Microscopic examination of peripheral blood smears of malaria positive smears for morphological abnormalities not only confirmed the presence of small platelet aggregates and giant platelets that triggered the platelet aggregate flag, but also revealed monocytes that contained malaria pigments as well as erythrophagocytosed infected red blood cells (Figures 3 and 4)

#### Platelet aggregation

Platelet aggregation refers to the clumping together of platelets in the blood and comprises homogenous clusters of 3-12 individual platelets. When present in the blood sample they are detected by the Analyzer on the basis of their size using a 256-channel pulse–height analyzer. Platelet aggregate flag is generated by the Analyzer when platelet aggregation is detected. Significantly more children in the malaria-infected group had platelet aggregation (25% vs. 4%, p<0.0001). Stratifying the data by different platelet count revealed an inverse relationship between platelet aggregation and platelet count irrespective of malaria infection status (Table 5). Moreover, difference was not significant between the number of children with thrombocytopaenia and platelet aggregation in the malaria infected group and that of thrombocytopaenic cases with platelet aggregates in non-malaria infected group (23% vs. 18%, *p=0.6*). Thirteen percent of children with normal platelet counts had platelet aggregation.

**Table 5:** Relationship between Platelet aggregate flag, platelet count

Platelet counts x 10^3^	Patient Category	Samples in the Group	Samples with Platelet aggregate flag	%
<150	Malaria Negative	16	3	18
	Malaria Positive	255	59	23
151-450	Malaria Negative	356	13	3.6
	Malaria Positive	258	73	28
>450	Malaria Negative	56	2	4
	Malaria Positive	10	1	10

### Cold agglutinin

Cold agglutinins refer to the circulating antibodies directed against own red blood cells and which bind to RBC at low temperatures. These are detected by the hematology analyzer and the cold agglutinin flag is generated. There was no relationship between the cold agglutinin detection and platelet count. The frequency of cold agglutinins in children with thrombocytopaenia irrespective of malaria infection status (42%) was comparable with that of children with normal platelet counts (36%). The presence of cold agglutinins was correlated with malaria infection as 64% of the children with cold agglutinins also had malaria (Table 6).

**Table 6:** Frequency of cold Agglutinin flag in the study groups

Platelet count X 10^3^ /µL	Patient category	samples in the group	Samples with cold agglutinin flag	(%)
<150	Malaria Negative	16	2	13
	Malaria Positive	255	112	44
150-450	Malaria Negative	356	95	27
	Malaria Positive	258	113	44
>450	Malaria Negative	56	31	55
	Malaria Positive	10	6	50
Totals		951	359	38

### White blood cells

Leucocytosis was defined as total white cell count>17,000/μL. Although leucocytosis was frequently seen in the malaria-infected group than the non-infected group (8% vs. 3%, *p=0.0028*), there was no significant difference in total WBC between the two groups (median 10,000/µL vs. 9100/µL, *p*=0.27). However leukocyte components were significantly affected. Neutrophil count (median 2,800/µL vs. 2,600/µL*, p=0.0076*), monocyte count (median 1,300/µL vs. 1,020/µL, *p*<0.0001) were all significantly elevated in the malaria-infected group as compared to the non-malaria infected group. Eosinophil count (median 140/µL vs. 230/µL, *p<0.0001*) and lymphocyte count (median 4200/µL vs. 4900/µL, *p=0.0009*) were significantly reduced in the malaria-infected group as compared to the non-infected group (Table 1). Malaria infected children were more likely to have monocytosis (defined as count >1.5 x 10^3^/uL) (39% vs. 16%, *p=0.0006*) compared to those not infected. Malaria infected children were also more likely to have lymphocytopenia defined as count <2.0 x 10^3^/uL compared to the non-infected ones (6% vs. 1.5%, *p=0.0016*). Monocyte count was positively correlated with parasite density (r=0.23, *p=0.0003*) and negatively with age (r=-0.15, *p=0.0042*). There was no demonstrable relationship between total WBC, neutrophil or lymphocyte counts with parasite densities or age.

## Diagnostic values of haematological parameters

Low lymphocyte, high WBC and high neutrophil counts had fairly good sensitivities but lacked specificity to accurately diagnose malaria. Low platelet count, low haemoglobin and high monocyte counts had better sensitivities and specificity for diagnosis of malaria. These parameters also had better odds ratios. Children with platelet<150,000/uL were up to 13.8 (OR) times more likely to have malaria than children with normal platelet (Table 7).

A combination of thrombocytopaenia and anaemia with high sensitivity (80%), specificity (84%) and odds ratio (22) makes it the best predictor of malaria (Table 8). Children with this combination are 22 times more likely to have malaria than children without this combination. A combination of thrombocytopaenia and monocytosis, (OR=15) though not very specific for malaria is also a useful indicator of malaria.

**Table 7:** Sensitivity, specificity, predictive value and odds ratio of the haematological parameters in diagnosis of malaria

*Variable*	Sensitivity*	Specificity*	PPV*	NPV*	*OR*	*LR*
	*(95% CI)*	*(95% CI)*	*(95% CI)*	*(95% CI)*	*(CI)*	
Platelets< 150000/µL	90 (86,95)	61 (57,66)	69 (64,73)	86 (84,89)	13.8 (7.2,24.8)	2.3
Hb<10g/dl	74 (69,78)	65 (61,68)	60 (55,64)	78 (74,81)	5 (3.9,6.7)	2.1
Monocytes count >1500/µL	74 (67,79)	54 (50,57)	38 (35,43)	84 (80,87)	3.34(2.5, 4.6)	1.6
Lymphocytes count<2000/µL	75(59,87)	46(43,49)	6(4,8)	97(95,98)	2.7(1.3,5.8)	1.4
WBC>17000/uL	70 (56,81)	46 (43,49)	8 (6,1)	96 (93,98)	2.1 (1.1,3.7)	1.3
Neutrophils count >7500/µL	72(60,82)	38(34,42)	10(7,12)	93(90,96)	1.6(0.9,2.8)	1.2

PPV- Positive Predictive Value, NPV-Negative Predictive value, LR- Likelihood ratio, *Percent, OR=odds ratio

**Table 8:** Sensitivity, Specificity, Predictive value and Odds ratio for combination of haematological parameters in diagnosis of malaria

Variable	sensitivity*	Specificity*	PPV*	NPV*	OR	LR
	(95% CI)	(95% CI)	(95% CI)	(95% CI)		
Monocyte count>1500/μL& Platelet<150000/uL	99 (86,99)	43(39,47)	12 (9,15)	98 (97,99)	15.3(5,49)	1.68
Platelet count <150000/μL & Hb<10g/dl	80 (72,86)	84 (76,91)	86(78,92)	78 (69,85)	22 (12,45)	5.2
Hb<10g/dl & Monocyte count >1500/uL	82 (75,87)	44 (40,48)	28 (24,32)	90(86,93)	3.6(2.4,5.6)	1.47

PPV- Positive Predictive Value, NPV-Negative Predictive value, OR- Odds ratio, LR Likelihood ratio *Values in percent

## Discussion

This study confirms that haematological abnormalities considered hallmark of malaria infection are common and more pronounced in *P. falciparum* malaria infection, probably due to the higher levels of parasitaemia found in these patients. The abnormalities previously reported include changes in haemoglobin, leucocyte count, platelet abnormalities resulting in defective thromboplastin, and disseminated intravascular coagulation (DIC) [8,11,14].

Anaemia is one of the most common complications in malaria especially in younger children and pregnant women in high transmission areas [15]. It is thought to result from a combination of haemolytic mechanisms and accelerated removal of both parasitized and non-parasitized red blood cells, depressed and ineffective erythropoiesis [11,16,17]. Abnormally high level of tumor necrosis factor (TNF), in malaria has been associated with marrow suppression [11]and imbalance in RBC surface markers such as CR1 [2].The present study, reports a significant reduction in Hb level in children infected with *P. falciparum* as compared to those not infected (Table 2). However, severe anaemia (Hb<5g/dl) was only seen in a minority (3%) of the children infected with malaria and none in the non-infected, most likely because this was a community-based study. The mild anaemia reported in 27% of the non-malaria infected children and some of the malaria-infected children may in part reflect poor nutritional status, background haemoglobinopathy, intestinal worm infestation and previous and/or repeated malaria infections in this area. Of these factors, haemoglobinophathies (HbAS, G6PD^-^ α^*^) could be the most important in the current study singly or in combination as shown in (Table 2) where 42% of the non-malaria infected children had one or more types of haemoglobinopathies compared to 25% of the non-anemic non-malaria infected children. Sickle cell trait incidence (28 %) [18] is common in Western Kenya. This compounded by high malaria transmission poses significant health problems in this region due to hereditary and acquired haemolytic anaemias although these children are protected from severe malaria disease.

In this study, although leucocytosis was frequently seen in the malaria-infected children, no significant difference in WBC was found between the two groups. In contrast, other studies have demonstrated leucopaenia [3,7,19] or leucocytosis [20,21]. These findings are comparable with those of other studies [17,22], which reported no significant difference in WBC between the malaria infected and non-infected groups. Monocytosis was the most important leukocytic change associated with malaria infection in this study with increased count being reported in the malaria infected group (median 1300/µL vs. 1020/µL, *p*<0.0001). Additionally monocyte count was positively associated with parasitaemia and negatively with age. The high monocyte count had been reported in patients with uncomplicated malaria [23] and subjects in this study also had uncomplicated malaria. However, this is in contrast to a previous study that reported low monocyte count being associated with severe malaria and an adverse outcome [21]. None of these studies reported an association between monocyte count with parasitaemia or age. As previously reported [17,21,23] children with malaria in this study had significantly high neutrophil count compared to the non-malaria infected children. While low lymphocyte count is not uncommon in malaria [3,11,14] the decrease in lymphocyte counts associated with malaria observed in this study are unusual. This finding may reflect redistribution of lymphocytes with sequestration in the spleen [11]. Lymphocytosis has also been reported elsewhere [21]. The observation of decreased eosinophil counts in this study is in agreement with previous studies [23,24]. This may suggest suppressed eosinophil production or release from the marrow or enhanced peripheral removal. There was no demonstrable relationship between leucocytosis, lymphocytopaenia or neutrophilia and parasite densities or age in this study. This may tend to support theory of host reaction to parasitaemia. Association between parasitaemia and haematological parameters should be interpreted with care because peripheral parasitaemia does not necessarily reflect the total body burden of parasites due to sequestered parasites, which still contribute to clinical manifestation [25].

Mononuclear cells, which are activated by *Plasmodium* during malarial attack, produce inflammatory cytokines, such as tumor necrosis factor (TNF), interleukin-1 and interleukin-6 (IL1, IL6). These cytokines stimulate the hepatic synthesis of acute phase inflammatory proteins, including CRP, which increase in malaria.

Phagocytosis of malaria pigments, infected and uninfected RBCs by monocytes, and neutrophils, has been observed in blood films of patients with malaria and is associated with disease severity [16,11,21,23]. The pigments are due to haemozoin from digested haemoglobin [25,26]. This study demonstrates that these changes occur early in the disease and not necessarily a marker of severe disease as some of the peripheral blood smears examined showed monocytes that contained malaria pigments and parasitized erythrocytes (Figure 3 and 4).

Platelet abnormalities in malaria are both qualitative and quantitative. In this study, platelet counts were significantly reduced in malaria-infected children. Thrombocytopaenia occurred in 49% of malaria cases and was inversely related to parasite density (Figure 2). Platelet count was the only parameter in the malaria-infected group that showed a decreasing trend across quartiles of parasite density (Table 4). Over 50% of the children with high parasitaemia (> 10%) had a platelet count of <50,000/uL. These observations imply that thrombocytopaenia may be a marker of parasite burden and disease severity. The association of platelet count and malaria has previously been described [23,27,28]. Two of our findings i.e., strong association with malaria and a trend across levels of parasite densities corroborates findings of a study of semi-immune population in Thailand [3]. Children with low platelet counts were also likely to have anaemia (r=0.2, *p=0.0003*) as previously reported from a study in Nigeria [20], which showed that baseline platelet counts were related to day twenty eight haematocrit, but no correlation between platelet counts and parasite densities. A South Korean study reported correlation between thrombocytopenia and a higher degree of *Plasmodium vivax* parasitaemia and increased cytokine production [29]. In the present study not only was the mean platelet volume significantly higher in children with malaria but was also inversely related to platelet counts. Mean MPV increased as platelet count decreased in both malaria infected and non-infected children (Figure 1). This may reflect an early release of platelets from the bone marrow in response to reduced platelet level in the body. The raised MPV can be explained by the giant platelets observed in some of the peripheral blood smears examined (Figures 3 and 4).

Thrombocytopaenia seems to occur through peripheral destruction [21], excessive removal of platelets by splenic pooling [19,30] as well as platelet consumption by the process of DIC [31]. This study confirms lack of bleeding in thrombocytopaenic malaria patients as previously reported [28]. Hypersensitive platelets, which are thought to enhance haemostatic responses, have been reported and may be the reason why bleeding episodes are rare in acute malaria infection, despite the thrombocytopaenia [28,32,33]. Reports of adequate or increased number of megakaryocytes in the bone marrow, makes decreased thrombopoiesis an unlikely cause of thrombocytopaenia in malaria [19]. Immune-mediated destruction of circulating platelets has been postulated as a cause of thrombocytopaenia seen in malaria. Platelets have also been shown to mediate clumping of *P. falciparum* infected erythrocytes [34]. This could lead to pseudo thrombocytopaenia. Malaria infected patients have elevated levels of specific IgG in their blood which binds to platelet-bound malaria antigens [33] possibly leading to accelerated destruction.

Platelet clumping was the most important platelet functional abnormality observed in this study. A large number of small platelets are seen mixed or clamped with a few giant platelets possibly due to the cytokine interference of megakaryopoiesis [35]. The findings in our study are consistent with these observations. Virtually all the peripheral blood smears from samples with platelet aggregate flag revealed small platelet aggregates mixed with giant platelets (platelets that approach or exceed the size of a red cell Figure 4), which most likely triggered the platelet aggregation. The platelets clumps made of groups of three to 12 platelets are falsely counted as single platelet by the analyzers thus causing pseudo-thrombocytopaenia. These observations suggest that, in as much as patients with malaria are likely to develop thrombocytopaenia, a reduced platelet count in some patients may be attributed in part to pseudo-thrombocytopaenia further explaining the lack of bleeding tendency. However, presence of giant platelets as well as increased MPV argues against pseudo-thrombocytopenia being the likeliest cause of thrombocytopaenia in malaria-infected individuals. Besides, a number of samples did not have microscopically detectable platelet aggregates despite having significant thrombocytopaenia. Giant platelets and increased MPV may indicate a compensatory premature release of platelets from the bone marrow [21] and would be consistent with true thrombocytopaenia occasioned by among others, peripheral destruction rather than pseudo-thrombocytopaenia.

Significantly more samples in the parasitaemic group had platelet aggregation suggesting that the aggregation was associated with malaria. However, when the data was stratified according to different platelet count ranges, a different pattern emerged suggesting that the platelet aggregation frequency was mainly related to platelet count rather than malaria infection status given that the number of thrombocytopaenic samples with the platelet aggregation in the malaria infected group increased in parallel with that of thrombocytopaenic samples in the non-malaria infected group (23% Vs. 18%, *p=0.6*) Table 5. However this should be interpreted with caution as there were only 16 children in the non-infected group with thrombocytopaenia and this being a malaria endemic area you may not rule out some of them recovering from a previous malaria episode. The finding of 73 cases (28%) in the malaria infected group with normal platelet counts having the platelet aggregation compared to only 13 samples (3.6%) in the non-malaria infected group further implies that this phenomenon may be largely related to malaria infection. The frequency of platelet aggregation was 8 times as higher in the malaria-infected children as opposed to non-infected group. This observation possibly rules out random factor related to the auto-analyzers or clots due to poor sampling technique as the causes of the flagging. Therefore, malaria infection is the most likely cause of either thrombocytopaenia or platelet aggregation. This is in agreement with findings of Scott *et al*[27]. there is no documented study so far that has established a clear cause of platelet clumping associated with malaria. Increased C reactive protein (CRP) is an indicator of inflammation and is associated with malaria [36]. It is thought to be responsible for the platelet clumping. It has been observed that platelet dysfunction resulting in hyperaggregation occurs in association with malaria [37].

The auto-analyzer also detected cold agglutinins which was more common in samples from malaria-infected children (64 %) (Table 6). However, platelet count did not correlate to the presence of cold agglutinins as the proportions were comparable in those children with thrombocytopaenia and those with normal platelet count (42% vs. 36% p=0.08). This study did not confirm the presence of the cold antibodies by a reference method such as Direct Coombs test (DAT) to determine their specificity or titers, however, it is assumed the cold agglutinins are related to malaria antigens binding to platelet-bound malaria antigens with the resultant platelet clumping observed... This may suggest an autoimmune reaction as another possible cause of malaria associated thrombocytopaenia. Elevated levels of platelet associated IgG have been observed in malaria patients with thrombocytopaenia [33]. A previous study in Gambian children [38] reported an association between high incidences DAT positivity and falciparum parasitaemia with raised antibody titers to falciparum schizonts. This is the first documentation to highlight the association of cold agglutinins as detected by haematology analyzers with malaria parasitaemia.

The present study demonstrates that low haemoglobin levels (OR=5) and low platelet counts (OR=13.8) are the two most reliable haematological parameters in predicting falciparum malaria in children from endemic areas. Children with thrombocytopaenia were 13.8 times more likely to have malaria than those with normal platelet count while anaemic children were 5 times more likely to have this outcome than non-anemic children. Thrombocytopaenia and anaemia with sensitivity of 90% and 74%, specificity 61% and 65% respectively, would result in use of anti-malarial drugs in 35%-39% of malaria negative cases, but would only result in 10-26% of true malaria cases not being treated (Table 7). This is an error level for over-treatment that may be acceptable compared to presumptive clinical diagnosis currently practiced in most malaria endemic areas. The findings are consistent with other studies [3,7,27,28], but none of these studies showed the predictive power of high monocyte count. This parameter with a sensitivity and specificity of 74% and 54% respectively had the best predictive value for malaria among all leucocytic cells. Children with increased monocyte count were three (OR=3) times more likely to have malaria than children with normal monocyte count. Low lymphocyte count, increased WBC and neutrophil counts had good sensitivities but lacked specificity to accurately diagnose malaria.

When haematological parameters were evaluated in combination, anaemia and thrombocytopaenia still had the greatest power in predicting malaria infection (Table 8). This study shows that a combination of low platelet count and Hb significantly increased the yield of diagnostic for malaria with sensitivity of 80%, specificity 84%. With this combination only 16% of malaria negative cases would receive anti-malarials and 20% of true malaria cases would not receive treatment. This combination also increases the probability of detecting malaria infection by up to 22 (95% CL 12-45) times in children from malaria endemic regions. A combination of low platelet count and high monocyte count also had a significant diagnostic value (sensitivity 99%, OR=15). In this malaria endemic area, a combination of the three parameters irrespective of clinical parameters like fever should always be re-evaluated for malaria especially in children that are symptomatic but have low density parasitaemia resulting in a false negative blood smear or rapid diagnostic test.

In conclusion, infection with *P falciparum* produces significant changes of haematological parameters in children living in malaria endemic regions. The most commonly affected parameters are platelets, haemoglobin, absolute monocyte counts and MPV. Thrombocytopaenia and monocytosis are associated with parasite density and, therefore, may be a marker of disease severity. The reduced platelet counts observed in some children may be related in part to pseudo-thrombocytopaenia but immune mediated thrombocytopaenia may not be ruled out. Although there was a trend towards platelet aggregation being associated with thrombocytopaenia but the frequency may still be primarily associated with malaria parasitaemia.

Presence of thrombocytopaenia in combination with anaemia and monocytosis in children from endemic areas may be useful as supportive diagnostic criteria for malaria in circumstances where definitive microscopic or RDT may be sub-optimal, as may be the case with low parasite density. Therefore, when used in addition to clinical and microscopy parameters, it can significantly improve malaria diagnosis and ideally prompt timely initiation of anti-malarial therapy. Further studies are required to characterize the cold agglutinins, platelet aggregates and explain their association with malaria.

The large sample size used in this study gives this analysis power to detect differences between malaria infected and non-malaria infected cases. Limitations include lack of previous medical history including anti-malarial treatment for the non-infected cases, which could potentially affect the interpretation of the results. Additionally no further investigations were done to rule out other infections such as bacterial and viral that could produce such haematological alterations since diseases such as HIV are associated with thrombocytopenia [39]. However, the children were relatively clinically stable.

## Competing interests

None of the authors had conflict of interests in the results of the study.

## Funding

Study was funded by the PATH-Malaria Vaccine Initiative, the Malaria Vaccine Development Program of the U.S. Agency for International Development, and the US Army Medical Research and Materiel Command.

## Disclaimer

The views of the authors do not necessarily reflect the position of the Kenya Medical Research Institute or the Department of the Army or the Department of Defense of USA.

## Authors Contribution

RNM – coordinated specimen collection and analysis of clinical specimens, statistical analysis, manuscript writing and participated in study design. GH – participated in specimen collection and oversight for malaria microscopy. JW- coordinated Hb typing. DW, CG, LO and DJ participated in data analysis and manuscript writing. BRO Conceived the study, participated in study design, data analysis, manuscript writing and was the lead study investigator. All authors read and approved the final manuscript.

## References

[B1] World Health Organization World malaria report 2008 Geneva

[B2] Waitumbi JN Opollo MO Muga RO Misore AO Stoute JA Red cell surface changes and erythrophagocytosis in children with severe *Plasmodium falciparum* anemia Blood 2000 95 1481 1486 10666228

[B3] Erhart LM Yingyuen K Chuanak N Buathong N Laoboonchai A Miller RS Meshnick SR Gasser RA Jr Wongsrichanalai C Hematological and clinical indices of malaria in a semi-immune population of Western Thailand Am J Trop Med Hyg 2004 70 8 14 14971691

[B4] Barnish G Bates I Iboro J Newer drug combinations for malaria BMJ 2004 328 1511 1512 15217846 10.1136/bmj.328.7455.1511 PMC437132

[B5] Hume JC Barnish G Mangal T Armázio L Streat E Bates I Household cost of malaria over diagnosis in rural Mozambique Malar J 2008 7 33 18282270 10.1186/1475-2875-7-33 PMC2279141

[B6] World Health Organization New perspective, malaria diagnosis Geneva 2000

[B7] Lathia TB Joshi R Can hematological parameters discriminate malaria from non malarious acute febrile illness in the tropics? Indian J Med Sci 2004 58 239 244 15226575

[B8] Reyburn H Mbakilwa H Mwangi R Mwerinde O Olomi R Drakeley C Whitty CJ Rapid diagnostic tests compared with malaria microscopy for guiding outpatient treatment of febrile illness in Tanzania randomized trial BMJ 2007 334 403 17259188 10.1136/bmj.39073.496829.AE PMC1804187

[B9] Wever PC Henskens YM Kager PA Dankert J Tom van Gool Detectetion of imported malaria with the cell –Dyn 4000 Hematology analyzer J Clin Microbiol 2002 40 4729 4731 12454179 10.1128/JCM.40.12.4729-4731.2002 PMC154588

[B10] Price RN Simpson JA Nosten F Luxemburger C Hkirjaroen ter Kuile F Chongsuphajaisiddhi T White NJ Factors contributing to anemia after uncomplicated falciparum malaria Am J Trop Med Hyg 2001 65 614 22 11716124 10.4269/ajtmh.2001.65.614 PMC4337986

[B11] Wickramasinghe SN Abdalla SH Blood and bone marrow changes in malaria Bailliere’s Clin Hematol 2000 13 Harcourt Pub Ltd 277 299 10.1053/beha.1999.0072 10942626

[B12] Ogutu BR Apollo OJ McKinney D Okoth W Siangla J Dubovsky F Tucker K Waitumbi JN Diggs C Wittes J Malkin E Leach A Soisson LA Milman JB Otieno L Holland CA Polhemus M Remich SA Ockenhouse CF Cohen J Ballou WR Martin SK Angov E Stewart VA Lyon JA Heppner DG Withers MR MSP-1 Malaria Vaccine Working Group Blood stage malaria vaccine eliciting high antigen specific antibody concentrations confers no protection to young children in Western Kenya PLoS ONE 2009 4 e4708 19262754 10.1371/journal.pone.0004708 PMC2650803

[B13] Ministry of Health National guidelines for diagnosis, treatment and prevention of malaria for health workers in Kenya Division of Malaria Control 2006 Ministry of Health. Kenya

[B14] Richards MW Behrens RH Doherty JF Hematological changes in acute, imported *Plasmodium falciparum* malaria Am J Trop Med Hyg 1998 59 859 9886188 10.4269/ajtmh.1998.59.859

[B15] Menendez C Fleming AF Alonso PL Malaria-related anaemia Parasitol Today 2000 16 469 476 10.1016/S0169-4758(00)01774-9 11063857

[B16] Weatherall DJ Miller LH Baruch DI Marsh K Doumbo OK Casals-Pascual C Roberts DJ Malaria and the red cell Hematology Am Soc Hematol Educ Program 2002 35 57 12446418 10.1182/asheducation-2002.1.35

[B17] Bashawri LA Mandil AA Bahnassy AA Ahmed MA Malaria: hematological aspects Ann Saudi Med 2002 22 372 376 17146269 10.5144/0256-4947.2002.372

[B18] Aluoch JR Higher resistance to Plasmodium falciparum infection in patients with homozygous sickle cell disease in Western Kenya Trop Med Int Health 1997 2 568 571 10.1046/j.1365-3156.1997.d01-322.x 9236824

[B19] Beale PJ Cormack JD Oldrey TB Thrombocytopenia in malaria with immunoglobulin (IgM) changes BMJ 1972 1 5796 345 349 5008661 10.1136/bmj.1.5796.345 PMC1787235

[B20] Adedapo AD Falade CO Kotila RT Ademowo GO Age as a risk factor for thrombocytopenia and anaemia in children treated for acute uncomplicated falciparum malaria J Vector Borne Dis 2007 44 266 271 18092534

[B21] Ladhani S Lowe B Cole AO Kowuondo K Newton CR Changes in white blood cells and platelets in children with falciparum malaria: relationship to disease outcome Br J Haematol 2002 119 839 847 10.1046/j.1365-2141.2002.03904.x 12437669

[B22] Chiwakata CB Hammer CJ Dietrich M High levels of inducible nitric oxide synthase mrna are associated with increased monocyte counts in blood and have a beneficial role in *Plasmodium falciparum* malaria Infect Immun 2000 68 394 399 10603415 10.1128/IAI.68.1.394-399.2000 PMC97148

[B23] Abdalla SH Peripheral blood and bone marrow leucocytes in Gambian children with malaria: numerical changes and evaluation of phagocytosis Ann Trop Paediatr 1988 8 250 258 2467614 10.1080/02724936.1988.11748582

[B24] Davis TM Ho M Supanaranond W Looareesuwan S Pukrittayakamee S White NJ Changes in peripheral blood eosinophil count in falciparum malaria Acta Trop 1991 48 243 246 10.1016/0001-706X(91)90052-L 1671625

[B25] Mujuzi G Magambo B Okech B Egwang TG Pigmented monocytes are negative correlates of protection against severe and complicated malaria in Ugandan children Am J Trop Med Hyg 2006 74 724 729 16687669

[B26] Hänscheid T Egan TJ Grobusch MP Hemozoin: from melantonin pigment to drug target, diagnostic tool, and immune modulator Lancet Infect Dis 2007 7 675 685 10.1016/S1473-3099(07)70238-4 17897610

[B27] Scott CS Van Zyl D Ho E Ruivo L Mendelow B Coetzer TL Thrombocytopenia in patients with malaria: automated analysis of optical platelet counts and platelet clumps with the cell Dyn CD4000 analyzers Clin Lab Haematol 2002 24 295 302 10.1046/j.1365-2257.2002.00466.x 12358891

[B28] Gérardin P Rogier C Ka AS Jouvencel P Brousse V Imbert P Prognostic value of thrombocytopenia in African children with falciparum malaria Am J Trop Med Hyg 2002 66 686 691 12224575 10.4269/ajtmh.2002.66.686

[B29] Park JW Park SH Yeom JS Huh AJ Cho YK Ahn JY Min GS Song GY Kim YA Ahn SY Woo SY Lee BE Ha EH Han HS Yoo K Seoh JY Serum cytokine profiles in patients with *Plasmodium vivax* malaria: a comparison between those who presented with and without thrombocytopenia Ann Trop Med Parasitol 2003 97 339 344 10.1179/000349803235002416 12831519

[B30] Skudowitz RB Katz J Lurie A Levin J Metz J Mechanisms of thrombocytopenia in malignant tertian malaria BMJ 1973 2 515 518 4714466 10.1136/bmj.2.5865.515 PMC1589556

[B31] Essien EM The circulating platelet in acute malaria infection Br J Haematol 1989 72 589 590 10.1111/j.1365-2141.1989.tb04329.x 2673332

[B32] Kelton JG Keystone J Moore J Denomme G Tozman E Glynn M Neame PB Gauldie J Jensen J Immune –mediated thrombocytopenia of malaria J Clin Invest 1983 71 832 826 6220030 10.1172/JCI110836 PMC436939

[B33] Moulin F Lesage F Legros A-H Maroga C Moussavou A Guyon P Marc E Gendrel D Thrombocytopenia and *Plasmodium falciparum* malaria in children with different exposures Arch Dis Child 2003 88 540 541 12765928 10.1136/adc.88.6.540 PMC1763122

[B34] Pain A Ferguson DJ Kai O Urban BC Lowe B Marsh K Roberts DJ Platelet-mediated clumping of *Plasmodium falciparum*-infected erythrocytes is a common adhesive phenotype and is associated with severe malaria Proc Natl Acad Sci U S A 2001 98 1805 1810 11172032 10.1073/pnas.98.4.1805 PMC29338

[B35] Kelkar DS Patnaik MM Joshi SR Malarial hematopathy J Assoc Physicians India 2004 52 611 612 15847352

[B36] Verhoef H West CE Kraaijenhagen R Nzyuko SM King R Mbandi MM van Laatum S Hogervorst R Schep C Kok FJ Malarial anemia leads to adequately increased erythropoiesis in asymptomatic Kenyan children Blood 2002 100 3489 3494 10.1182/blood-2001-12-0228 12393621

[B37] Mohanty D Marwaha N Ghosh K Sharma S Garewal G Shah S Devi S Das KC Functional and ultrastructural changes of platelets in malarial infection Trans R Soc Trop Med Hyg 1988 82 369 375 10.1016/0035-9203(88)90122-8 3068847

[B38] Facer CA Direct Coombs antiglobulin reactions in Gambian children with *Plasmodium falciparum* malaria. II. Specificity of erythrocyte-bound IgG Clin Exp Immunol 1980 39 279 288 6993068 PMC1538045

[B39] Casals-Pascual C Kai O Newton CR Peshu N Roberts DJ Thrombocytopenia in falciparum malaria is associated with high concentrations of IL-10 Am J Trop Med Hyg 2006 75 434 436 16968917

